# Rapid detection of antimicrobial residues in broiler meat using flow cytometry-driven multiplex immunoassay

**DOI:** 10.3389/fvets.2025.1636223

**Published:** 2025-10-30

**Authors:** Francesca Tiziana Cannizzo, Federica Sini, Sara Divari, Matteo Cuccato, Alessia Poggi, Sara Panseri, Maria Nobile, Luca Maria Chiesa, Fulvio Riondato

**Affiliations:** ^1^Department of Veterinary Science, University of Turin, Grugliasco, Italy; ^2^Department of Veterinary Medicine and Animal Science, University of Milan, Lodi, Italy

**Keywords:** Beadyplex, antimicrobials, immunoassay, flow cytometry, HPLC-HRMS

## Abstract

**Introduction:**

The misuse of antimicrobials (AMs) in poultry production contributes significantly to the global threat of antimicrobial resistance. Despite legislative efforts within the European Union (EU) to reduce AM use, Italy remains among the countries with the highest consumption levels. Effective and accessible screening tools for AMs residues in poultry meat are crucial for food safety monitoring and regulatory compliance. This study evaluates the performance of the Beadyplex flow cytometric assay as a field-applicable method to detect residues of thiamphenicol, sulphadiazine, and amoxicillin in broiler chicken skeletal muscle.

**Methods:**

*Pectoralis major* muscle samples were collected from 41 male broiler chickens (Ross 308) previously treated with thiamphenicol (*n* = 11), sulphadiazine (*n* = 6), amoxicillin (*n* = 12), or untreated (controls, *n* = 12). Beadyplex assays were performed following manufacturer instructions, and results were acquired using a standard flow cytometer. Sensitivity, specificity, predictive values, and kappa agreement were calculated to assess the performance of the test in recognizing the AMs treatment. The agreement with the current reference method for detecting AM residues (high-performance liquid chromatography–high-resolution mass spectrometry, or HPLC-HRMS) was also calculated.

**Results:**

The Beadyplex assay demonstrated high sensitivity and specificity for thiamphenicol (Se = 1.00; Sp = 0.83; *k* = 0.791) and sulphadiazine (Se = 1.00; Sp = 0.91; k = 0.813), with substantial to almost perfect agreement with HPLC-HRMS results (*k* = 0.706 and 0.827, respectively). Amoxicillin residues were not detected in any treated sample by Beadyplex.

**Discussion:**

The Beadyplex assay offers a promising, cost-effective, and rapid screening complementary to confirmatory analysis for detecting selected AM residues in poultry meat. Its substantial agreement with reference methods and ease of use with standard cytometers support its potential application in field-based veterinary surveillance and food safety programs.

## 1 Introduction

According to the FAO, chicken meat was the most produced worldwide in 2022 (1). The intensive farming system often relied on the use of antimicrobials (AMs) to prevent and treat diseases, and in some cases AMs are illegally used as growth promoters to improve meat production (2). According to the Thirteenth Report of the European Surveillance of Veterinary Antimicrobial Consumption (2018–2023) of the European Medicines Agency (EMA), in Italy the most commonly used AMs in food-producing animals belong to the following classes, respectively: penicillins, tetracyclines, and sulfonamides (3). Between 2018 and 2023, the consumption of veterinary AMs in food-production animals decreased by 26.11% in Italy. Moreover, many companies have adopted AMs-free production chains and certifications, awarded only when no AMs are used at any stage of the production (4). Despite these advancements, Italy remains the European country with the highest levels of AMs use in animal husbandry: 180.3 mg/PCU (3). As a result of their widespread misuse, antimicrobial resistance has become a priority issue worldwide (5–7).

To address this problem the European Union (EU) banned the use of AMs as growth promoters (Regulation (EC) 1831/2003) (8), and limited the prophylactic use to exceptional cases (Regulation (EU) 2019/6) (9). Furthermore, in the case of drug administration the EU requires a withdrawal period before broilers are processed for human consumption (Regulation (EC) 470/2009) (10), and has established Maximum Residue Levels (MRLs) for most AMs in various animal tissues and products (Regulation (EU) 37/2010) (11). In poultry, skeletal muscle represents one of the matrixes investigated, followed by eggs, liver, kidney, and skin (11). Conversely, the frequency of sampling is influenced by the extent and characteristics of local production in each European country. For example, in Italy, 5,740 meat samples were analyzed according to the “Piano Nazionale Residui 2024” report (12). High-performance liquid chromatography coupled with high-resolution mass spectrometry (HPLC-HRMS) is the official method approved by law for detecting AMs residues and ensuring compliance with MRLs, in accordance with Commission Implementing Regulation (EU) 2021/808 of 22 March 2021 (13). HPLC-HRMS is costly and time-consuming and requires specialized equipment and trained personnel. Faster and more cost-effective tests for screening samples before undergoing confirmatory analysis would be extremely useful. Several methods, such as immunoassay-based tests (ELISA or Lateral flow assays), microbial inhibition tests, and biosensor assays have been developed (14, 15). Unfortunately, these methods are limited to a few AMs, require sample preparation and clean-up to remove interfering substances, and need specific environmental conditions and handling. Finally, they are often expensive to produce and implement. Recently, multiplex immunoassays for the simultaneous detection of different AMs family have become available.

In particular, a Belgian company has developed and marketed a kit for the simultaneous detection of up to 10 families of AMs using flow cytometry (Beadyplex, Unisensor). The main advantage of the assay stems from the high multiplexing capacity (it covers 80 a.m. residues in one assay), resulting in time savings and reduced costs. According to the manufacturer, a wide range of matrices can be analysed (meat, milk, eggs, seafood), and the only necessary equipment is a flow cytometer with blue and red lasers and standard fluorescence channels. However, the analysis of samples and the interpretation of results have been validated by Unisensor with the use of a specific flow cytometer and dedicated software. In the absence of this specific equipment, the company offers a service for analyzing the Beadyplex plates. Despite its commercial availability, the Beadyplex assay remains underexplored in the peer-reviewed literature. To date, only a few conference abstracts and presentations have addressed its use in animal-derived products for screening AM residues (16, 17). Similar approaches have been applied to honey and milk samples with the aim of detecting AMs contamination (18, 19). The present study aimed to evaluate the effectiveness of the Beadyplex assay in detecting thiamphenicol, sulphadiazine, and amoxicillin residues in broiler meat. In addition, we present an alternative analytical workflow that allows for the use of the Beadyplex kit without relying on propriety instrumentation or the company’s analysis service, thereby providing a more accessible and cost-effective solution for screening AMs residues in poultry production.

## 2 Materials and methods

### 2.1 Animals and sample collection

Samples of pectoralis major muscle were collected at the time of slaughter during a previous zootechnical trial, as described in Giannuzzi et al. (20), and stored at −80 °C in liquid nitrogen until processing for the present study. This trial was approved by the Ethics and Animal Welfare Committee of the Department of Veterinary Sciences (protocol n. 275/22), University of Turin (Italy). The broiler rearing conditions were previously described by Cuccato et al. (21). Briefly, broilers housed in different pens were, respectively, treated with amoxicillin (group A; *n* = 12, amoxicillin 30 mg/kg BW twice/day), thiamphenicol (group T; *n* = 11, thiamphenicol 65 mg/kg BW once/day), and sulphadiazine (group S; *n* = 6, sulphadiazine 20 mg/kg BW once/day) according to a prophylactic program with administration during the starter period (from 0 to 25th broiler day, 3 consecutive days for amoxicillin and thiamphenicol, 5 days for sulphadiazine) and the finisher period (from 26th to 58th broiler day, 3 consecutive days for amoxicillin and thiamphenicol, 5 days for sulphadiazine). Animals of an additional pen were not treated and served as controls (group K, *n* = 12). Chickens were regularly slaughtered after the withdrawal periods provided by law. The samples of pectoralis major muscle (about one gram each) were analyzed for AMs content using flow cytometry and HPLC-HRMS.

### 2.2 Beadyplex assay and flow cytometric analysis

**Figure 1:**
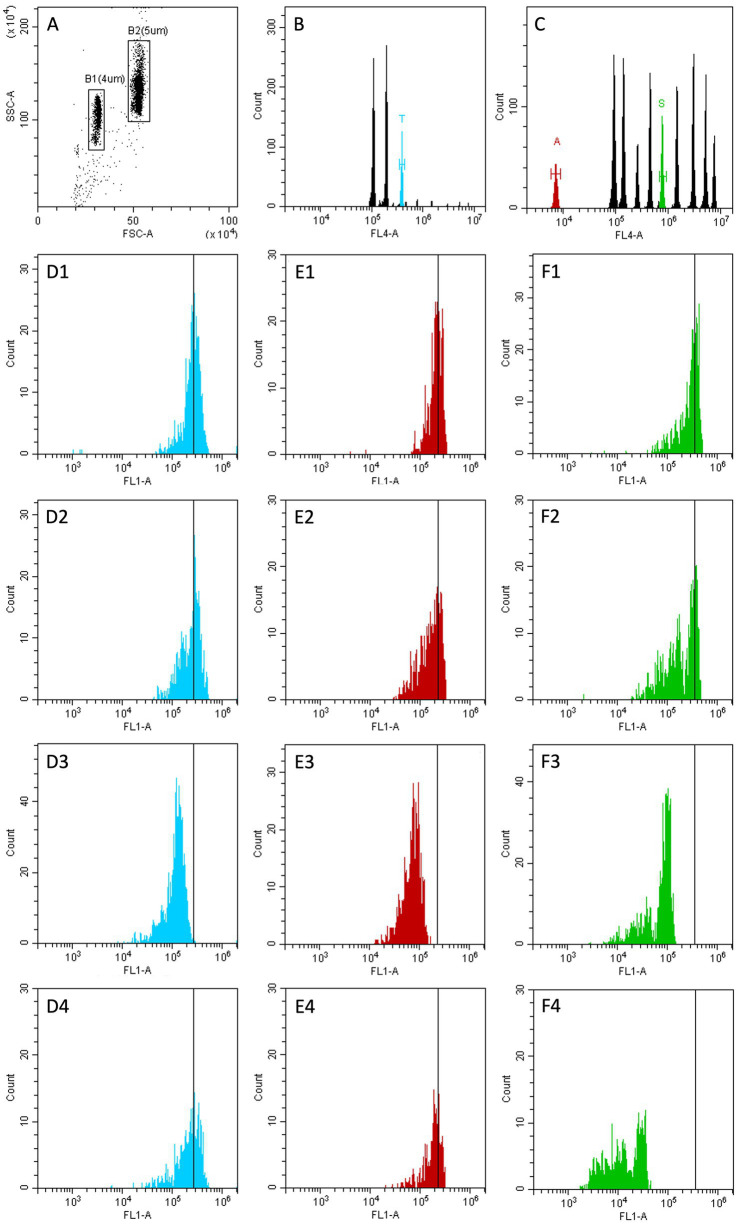
Gating strategy. **(A)** FSC vs. SSC plot after doublets exclusion. Two gates are depicted to isolate the microbeads populations (B1,B2). **(B)** only events in gate B1 are included. B1 population is divided in 3 subpopulations according to FL4 fluorescence. A region is set on the subset that binds thiamphenicol (T, light blue peak). **(C)** only events in gate B2 are included. B2 population is divided in 10 subpopulations according to FL4 fluorescence. Two regions are set on the subsets that binds amoxicillin (A, red peak) and sulphadiazine (S, green peak). **(D1–D4)** only events in region T are included. **(E1–E4)** only events in region A are included. **(F1–F4)** only events in region S are included. **(D1,E1,F1)** negative control. **(D2,E2,F2)** sample from group K (untreated animals). **(D3,E3,F3)** positive control. **(D4,E4,F4)** sample from group S (sulphadiazine treated animals).

Beadyplex assay was performed according to the manufacturer’s protocol. In brief, 1 mL of extraction buffer was added to 1 g of chicken skeletal muscle previously homogenized using the extraction buffer. After 10 min of agitation, tubes were centrifuged and 200 μL of supernatant were added into the pre-wetted sample filter plate. Filtered sample (50 μL) was transferred to the assay filter plate, beads mix and primary binders were added and, after 30 min of agitation, the plate was washed. A secondary binder was added and after incubation (15 min under agitation) the plate was washed. Each washing step was repeated 3 times and consisted in adding assay buffer and filtering using a vacuum manifold (MultiScreen HTS, Millipore, Merck KGaA, Darmstadt, Germania). Finally, 150 μL of assay buffer were added to each well and the plate was acquired at the flow cytometer. Two negative and one positive control were also processed as described. The positive control was prepared by adding the positive standard provided with the kit to a chicken muscle sample from an additional untreated animal. Samples were acquired with a Cytoflex cytometer (Beckman Coulter) equipped with a plate loader and data were analyzed with CytExpert software. Thiamphenicol, amoxicillin and sulphadiazine-specific beads were gated according to their size and primary fluorescence (FL4: red laser, 660/10 band pass filter). The mean fluorescence intensity (MFI) of the secondary signal (FL1: blue laser, 524/40 band pass filter) was recorded for each subset. As this is a competitive immunoassay, the presence of AM residues in the sample is indicated by a reduction in MFI. Detailed gating strategy is reported in Figure 1.

A shift to the left of FL1 fluorescence is a positive result (D3, E3, F3, F4): the presence of AM residues competes with the bead-specific binder conjugated with the fluorochrome leading to a reduction of the fluorescence signal (recorded as Mean Fluorescence Intensity, MFI).

### 2.3 HPLC-HRMS analyses

Two samples (1 each from groups T and A) were no longer available at the time of the analysis, which was performed on 27 cases. The extraction of AM residues from muscle was performed following the protocol by Chiesa et al. (22). Briefly, 1 g of muscle was spiked with the internal standard (IS) at 2 ng g − 1, extracted with 5 mL of McIlvaine buffer (pH 4.0) and 100 μL of 20% w/v of Trichloroacetic acid were added for protein precipitation. After centrifugation (2,500 × g, 4 °C, 10 min), the supernatant was defatted with 2 × 3 mL of n-hexane and an Oasis HLB cartridge preconditioned with 3 mL of methanol and 3 mL of Milli-Q water, was used for purification of the sample. After loading, the cartridge was washed with 2 × 3 mL methanol: water (5:95 v/v) and finally 5 mL of methanol was added for the compound elution. The eluate was evaporated and reconstituted in 200 μL of methanol:0,1%formic acid (10,90 v/v).

An HPLC-HRMS system (Thermo Fisher Scientific, San Jose, CA, USA) coupled to a Q-Exactive Orbitrap detector (Thermo Fisher Scientific) was used and the chromatographic condition and mass parameters are reported in the study of Chiesa et al. (22), while the method was validated according to the guidelines reported in the Commission Decision 657/2002/CE repealed by Implementing Regulation (EU) 2021/808 (13).

### 2.4 Statistical analysis

Statistical analysis was run on Analyse-It software (Analyse-It, Leeds, UK). MFI value was normalized (nMFI) as follow: nMFI = (MFIs/MFIc)*100, where MFIs is MFI measured in the sample and MFIc is the mean of MFI measured in the two negative controls.

The limit of detection (LOD) of the method was calculated for each AMs as the mean of the nMFI of all controls (group K) minus 3 standard deviations (23, 24). A nMFI below the LOD was defined as a positive result (AMs residue detected). Cohen’s kappa (*k*) agreement between Beadyplex results and treatment and between Beadyplex and HPLC-HRMS results was calculated for the animals in the T, A, and S groups and interpreted according to Landis and Koch (25) as follow: poor (0–0.20), fair (0.21–0.40), moderate (0.41–0.60), substantial (0.61–0.80) almost perfect (0.81–1.00). Contingency tables were designed for each AMs and used to evaluate the performance of the Beadyplex assay in detecting the AMs treatment, and reported as accuracy, specificity (Sp), sensitivity (Se), positive (PPV) and negative (NPV) predictive values, and positive (LR+) and negative (LR-) likelihood ratio. The administration of the AMs (yes vs. no) was used as reference result. As an example, in the thiamphenicol 2×2 table, the true positives are the thiamphenicol-treated animals while the true negatives are the amoxicillin and sulphadiazine treated animals.

## 3 Results

### 3.1 nMFI, LOD, HPLC-HRMS and K agreement analyses

nMFI values obtained from control animals and the calculated LOD values are reported in Supplementary Table S1. HPLC-HRMS analysis confirmed all control animals were negative for residues of thiamphenicol, amoxicillin and sulphadiazine. The nMFI values and interpretation for thiamphenicol, amoxicillin and sulphadiazine, along with the corresponding HPLC-HRMS results for each sample are detailed in Supplementary Tables S2–S4 (group T, A, and S, respectively). A summary of the results, comparing the treatment groups, is reported in contingency tables (Tables 1–3).

**Table 1:** Thiamphenicol: Beadyplex results compared to treatment.

		Treatment		Total
Beadyplex		POS	NEG	
	POS	11	3	14
	NEG	0	15	15
Total		11	18	29

Treatment POS and NEG are animals in group T (treated with thiamphenicol) and in groups A + S (treated with amoxicillin or sulphadiazine), respectively.

**Table 2:** Amoxicillin: Beadyplex results compared to treatment.

		Treatment		Total
Beadyplex		POS	NEG	
	POS	0	3	3
	NEG	12	14	26
Total		12	17	29

Treatment POS and NEG are animals in group A (treated with amoxicillin) and in groups T + S (treated with thiamphenicol or sulphadiazine), respectively.

**Table 3:** Sulphadiazine: Beadyplex results compared to treatment.

		Treatment		Total
Beadyplex		POS	NEG	
	POS	6	2	8
	NEG	0	21	21
Total		6	23	29

Treatment POS and NEG are animals in group S (treated with sulphadiazine) and in groups T + A (treated with thiamphenicol or amoxicillin), respectively.

Thiamphenicol residues were detected by the Beadyplex assay in 14 animals. Of these, all the 11 thiamphenicol-treated animals were positive, while 3 animals from groups A (2) and S (1) were also positive by Beadyplex but negative by HPLC-HRMS analyses. The assay performance in detecting thiamphenicol treatment was as follows: accuracy = 0.897, Se = 1, Sp = 0.833, PPV = 0.786, NPV = 1, LR + = 6, LR- = 0 (undefined). Agreement between Beadyplex results and *in vivo* treatment was substantial (*k* = 0.791).

The HPLC-HRMS analysis confirmed the presence of thiamphenicol residues in all treated cases (10/10; one sample was not available) while it was negative in all the untreated cases, with the exception of 2 broilers from group A (both negative at Beadyplex assay). All the samples from the control group were negative by HPLC-HRMS analysis. Overall agreement between Beadyplex and HPLC-HRMS results was substantial (*k* = 0.706).

The Beadyplex assay reported 3 amoxicillin-positive cases, all in group T. No animal in group A tested positive. Given these findings, statistical analysis for diagnostic performance was not run.

The HPLC-HRMS analysis did not detect amoxicillin residues in any sample except one in group A (very low concentration; data not shown). The agreement between Beadyplex and HPLC-HRMS results was almost perfect (*k* = 0.897).

The Beadyplex assay detected sulphadiazine residues in 8 animals. Of these, 6 positive samples belong to the sulphadiazine-treated animals (group S). The other 2 cases were found in group T, and they were among the 3 instances that showed false-positive results for amoxicillin.

The Beadyplex performance in detecting sulphadiazine treatment was as follows: accuracy = 0.931, Se = 1, Sp = 0.913, PPV = 0.75, NPV = 1, LR + = 11.5, LR- = 0 (undefined). Agreement between Beadyplex results and treatment was almost perfect (*k* = 0.813).

The HPLC-HRMS analysis confirmed the presence of sulphadiazine residues in all treated animals while it was negative in all untreated cases. Agreement between Beadyplex and HPLC-HRMS results was almost perfect (*k* = 0.827). LOD values have also been calculated for the other AM classes detected by the assay. Samples from treated animals showed an nMFI < LOD in 3 cases: 1 for chloramphenicol and 2 for *β*-lactams (data not shown). These three samples are the same ones that also showed false-positive results for amoxicillin and/or.

## 4 Discussion

The three AMs investigated in this study, amoxicillin, thiamphenicol, and sulphadiazine, are of widely used in veterinary medicine, particularly in treating infections in poultry. Amoxicillin, a penicillin-class AM, is widely used for its broad spectrum against bacterial pathogens (26). According to the Antimicrobial Advice *Ad Hoc* Expert Group (AMEG) classification of AMs for veterinary medicine, amoxicillin is categorized as a Group D AMs (“Use with caution”), meaning it should be used when other first-line alternatives are not appropriate or ineffective (27). However, the World Health Organization (WHO), in its AWaRe (Access, Watch and Reserve) classification, places amoxicillin in the Access group, identifying it as a critically important AM for human health, recognizing it as a first-choice AM with a low risk of resistance selection (28).

Alternatively, thiamphenicol, a derivative of chloramphenicol, belongs to the amphenicols class and its use was reintroduced after the ban of chloramphenicol in poultry (29). Thiamphenicol is classified by the EMA as belonging to Group C (“Cautious use”) (27). This AM is indicated when Group D treatments are ineffective and is mainly used for the treatment of serious infections. Although thiamphenicol is not directly listed in the AWaRe classification by the WHO, chloramphenicol, its analogue, is considered a highly important AM for human health and is included in the critically important antimicrobials (CIA) list (28).

Finally, sulphadiazine, an intermediate-acting sulfonamide, is used for the treatment of bacterial and protozoal (i.e., *Eimeria* spp.) infections in poultry (30). In the AMEG classification, it also falls into Group D (“Use with caution”), considered safe for veterinary use, but with limitations to reduce the risk of resistance (27). The WHO classifies it as a highly important AM for human health and includes it in the CIA list, although sulphadiazine is not explicitly included in the AWaRe classification (28).

These AMs, while widely used in veterinary medicine, are also associate with a risk of introducing bacterial resistance, which makes prudent and regulated use essential (31). The AMEG and CIA classifications highlight their therapeutic value for animal health, but also their potential impact on public health in cases of misuse.

In this regulatory context, there is a growing need for the development of new diagnostic tools to detect the illicit or improper use of AMs, offering faster monitoring than HPLC-HRMS analysis. The Beadyplex assay by Unisensor is a flow cytometric tool designed for the detection of AMs, suitable for use in various settings, including food safety and environmental monitoring. According to the manufacturer’s guidelines, specific instrumentation and software must be available to read the samples and interpret results. Alternatively, after processing the samples, the plate can be shipped to Unisensor for results analysis. Although the product has been on the market for several years, no field studies evaluating its use in samples from AMs treated animals have been published to date.

In this study, we provide an approach that allows independent analysis without the need for the specific instrument and software, thereby significantly expanding the applicability of the commercial kit. Moreover, we evaluate the assay’s ability to discriminate between thiamphenicol, amoxicillin and sulphadiazine-treated animals and untreated broilers.

As anticipated, the Beadyplex assay did not detect any positive samples in the amoxicillin-treated group, likely due to the drug’s pharmacokinetic properties. The depletion times of amoxicillin have been shown to be longer in the liver and kidneys than in muscle (26). Amoxicillin is known to leave less residues in muscle tissue, a finding confirmed by HPLC-HRMS analysis in our study. Further testing with other matrices, such as liver and kidney, which are known to be better targets for amoxicillin detection, should be considered to evaluate the potential of the assay for amoxicillin-treated animals (32).

On the contrary, the assay showed high accuracy in detecting both thiamphenicol and sulphadiazine treatment. All treated animals were correctly identified in front of few false-positive results.

Predictive values and LR values suggest that the assay is effective in discriminating between treated and untreated animals. In particular, the absence of false-negative results supports its potential as screening test limiting the need for HPLC-HRMS analysis to only positive samples. In addition to confirming positive results, Commission Implementing Regulation (EU) 2021/808 requires that confirmatory methods for detecting residues of pharmacologically active substances in food of animal origin must provide unequivocal identification on the analyte’s chemical structure (13). Accordingly, HPLC-HRMS must be coupled with appropriate detection systems, such as HRMS, tandem mass spectrometry (MS/MS), diode-array detection (DAD), or fluorescence detection. These levels must comply with the MRL established for market entry (Regulation (EU) 37/2010) (11). Importantly, HPLC-HRMS analysis confirmed that AMs residues were below the MRL in all treated broilers, thus confirming that the adhering to withdrawal periods allows the production of meat that complies with legal limits.

By generating a calibration curve, the Beadyplex assay can also provide quantitative results. However, evaluating this potential was beyond the scope of the current study, and its ability to detect residues above or below MRL remains to be tested. Considering its cost-effectiveness and rapid turnaround, the Beadyplex test demonstrates an applicability comparable to that of previously reported immunoassay- and biosensor-based approaches (14, 15). The main advantage of Beadyplex test is the ability to detect multiple AMs residues, while other rapid testing methods frequently detect only one AM molecule or a single AM class. For example, the rapid test evaluated by Meklati and colleagues is designed only for the detection of β-lactams or tetracyclines (33). In other cases, single AM molecules can be detected by investigated rapid tests, such as chloramphenicol or kanamycin (19, 34).

A key limitation of the described approach is the need for each laboratory to calculate its own nMFI values and LOD for each batch of the kit. However, this last pitfall can be easily addressed by freezing meat aliquots from untreated animals. The Beadyplex test may serve as a screening tool to identify negative samples, thereby reducing reliance on the gold standard method. Only suspected or positive samples would subsequently undergo HPLC-HRMS analysis to confirm the presence of antimicrobial residues. Such a testing strategy has the potential to lower the costs of official controls, particularly since the majority of samples are consistently reported (12).

## 5 Conclusion

To the best of the authors’ knowledge, this is the first study to report the application of the Beadyplex assay on animals treated under field conditions. The results obtained suggest that the assay can be used to effectively detect the presence of thiamphenicol and sulphadiazine residues, regardless of concentration, once the specific LOD is calculated. Although further studies are needed to explore different matrices, molecules, and potential interferences, our results highlight the promising potential of the Beadyplex assay as a screening method to complement confirmatory analyses in verifying MRL compliance and certifying AMs-free products.

## Data Availability

The original contributions presented in the study are included in the article/Supplementary material, further inquiries can be directed to the corresponding author.

## References

[1] Food and Agriculture Organization of the United Nations (FAO). World Food and Agriculture - Statistical Yearbook 2024. In: Chapter 2: Production, trade and prices of commodities. Rome: FAO (2024). pp. 181–185.

[2] Mehdi Y Létourneau-Montminy MP Gaucher ML Chorfi Y Suresh G Rouissi T. Use of antibiotics in broiler production: global impacts and alternatives. Animal Nutrition. (2018) 4:170–8. doi: 10.1016/j.aninu.2018.03.002, PMID: 30140756 PMC6103476

[3] European Medicines Agency. European sales and use of antimicrobials for veterinary medicine: Annual surveillance report for 2023. LU: Publications Office (2025).

[4] Mohammadi H Saghaian S Boccia F. Antibiotic-free poultry meat consumption and its determinants. Foods. (2023) 12:1776. doi: 10.3390/foods12091776 PMC10177776 37174314

[5] Dasenaki ME Thomaidis NS. Multi-residue methodology for the determination of 16 coccidiostats in animal tissues and eggs by hydrophilic interaction liquid chromatography – tandem mass spectrometry. Food Chem. (2019) 275:668–80. doi: 10.1016/j.foodchem.2018.09.138, PMID: 30724247

[6] Gonzalez Ronquillo M Angeles Hernandez JC. Antibiotic and synthetic growth promoters in animal diets: review of impact and analytical methods. Food Control. (2017) 72:255–67. doi: 10.1016/j.foodcont.2016.03.001

[7] Brown K Uwiera RRE Kalmokoff ML Brooks SPJ Douglas Inglis G. Antimicrobial growth promoter use in livestock: a requirement to understand their modes of action to develop effective alternatives. Int J Antimicrob Agents. (2017) 49:12–24. doi: 10.1016/j.ijantimicag.2016.08.006, PMID: 27717740

[8] European Union. Regulation (EC) No 1831/2003 of the European Parliament and of the Council of 22 September 2003 on additives for use in animal nutrition. *Official J Euro Union* (2003) 268:29–43.

[9] European Union. Regulation (EC) No 470/2009 of the European Parliament and of the Council of 6 May 2009 laying down Community procedures for the establishment of residue limits of pharmacologically active substances in foodstuffs of animal origin, repealing Council Regulation (EEC) No 2377/90 and amending Directive 2001/82/EC of the European Parliament and of the Council and Regulation (EC) No 726/2004 of the European Parliament and of the Council. *Official J Euro Union* (2009) 152:11–22 (2009).

[10] European Commission. Commission Regulation (EU) No 37/2010 of 22 December 2009 on pharmacologically active substances and their classification regarding maximum residue limits in foodstuffs of animal origin. Official J Euro Union (2010) 15:1–72.

[11] European Union. Regulation (EU) 2019/6 of the European Parliament and of the Council of 11 December 2018 on veterinary medicinal products and repealing Directive 2001/82/EC. Official J Euro Union (2019) 4:43–167.

[12] Ministero della Salute. Piano Nazionale per la ricerca dei Residui (PNR) – Relazione annuale 2024. Roma: Ministero della Salute (2025).

[13] European Commission. Regulation (EU) 2021/808 of 22 March 2021 on the performance of analytical methods for residues of pharmacologically active substances used in food-producing animals and on the interpretation of results, as well as on the methods to be used for sampling and repealing Decisions 2002/657/EC and 98/179/EC (Text with EEA relevance). Official J Euro Union (2021) 180, 84–109.

[14] Getahun M Abebe RB Sendekie AK Woldeyohanis AE Kasahun AE. Evaluation of antibiotics residues in Milk and meat using different analytical methods. Int J Analytical Chemistry. (2023) 2023:1–13. doi: 10.1155/2023/4380261, PMID: 37424721 PMC10328735

[15] Frigoli M Krupa MP Hooyberghs G Lowdon JW Cleij TJ Diliën H. Electrochemical sensors for antibiotic detection: a focused review with a brief overview of commercial technologies. Sensors. (2024) 24:5576. doi: 10.3390/s24175576, PMID: 39275486 PMC11398233

[16] Suarez Pantaleon C Gallo A Chabottaux V Huet A-C Brasseur A Delahaut P. Beadyplex: A novel MULTI-antibiotic flow cytometric screening method for food commodities. Prague: Czech Republic (2015).

[17] Suarez Pantaleon C. Validation of the flow cytometry immunoassay, Beadyplextm, for Multi-class and Multi-residue screening for antibiotics in fish. IAFP (2017). Available online at: https://iafp.confex.com/iafp/euro17/webprogram/Paper15005.html (Accessed May 21, 2025).

[18] Muriano A Chabottaux V Diserens J-M Granier B Sanchez-Baeza F Marco M-P. Rapid immunochemical analysis of the sulfonamide-sugar conjugated fraction of antibiotic contaminated honey samples. Food Chem. (2015) 178:156–63. doi: 10.1016/j.foodchem.2015.01.037, PMID: 25704696

[19] Zhao M Li X Zhang Y Wang Y Wang B Zheng L. Rapid quantitative detection of chloramphenicol in milk by microfluidic immunoassay. Food Chem. (2021) 339:127857. doi: 10.1016/j.foodchem.2020.127857, PMID: 32866699

[20] Giannuzzi D Biolatti B Longato E Divari S Cucuzza LS Pregel P. Application of RNA-sequencing to identify biomarkers in broiler chickens prophylactic administered with antimicrobial agents. Animal. (2021) 15:100113. doi: 10.1016/j.animal.2020.100113, PMID: 33573988

[21] Cuccato M Scaglione FE Centelleghe C Divari S Biolatti B Pregel P. Assessment of antimicrobial effects on broiler gut barrier through histopathology and immunohistochemistry of tight-junction proteins. Front Vet Sci. (2022) 9:830073. doi: 10.3389/fvets.2022.830073, PMID: 35425830 PMC9002056

[22] Chiesa LM Nobile M Panseri S Arioli F. Suitability of feathers as control matrix for antimicrobial treatments detection compared to muscle and liver of broilers. Food Control. (2018) 91:268–75. doi: 10.1016/j.foodcont.2018.04.002

[23] O’Hara DM Xu Y Liang Z Reddy MP Wu DY Litwin V. Recommendations for the validation of flow cytometric testing during drug development: II assays. J Immunol Methods. (2011) 363:120–34. doi: 10.1016/j.jim.2010.09.036 20946898

[24] Wood B Jevremovic D MC B e e Yan M Jacobs P Litwin B. Validation of cell-based Fluo- rescence assays: practice guidelines from the ICSH and ICCS – part V – assay performance criteria. Cytometry B Clin Cytom. (2013) 84B:315–23. doi: 10.1002/cyto.b.21108 24022854

[25] Landis JR Koch GG. The measurement of observer agreement for categorical data. Biometrics. (1977) 33:159. doi: 10.2307/2529310, PMID: 843571

[26] Zhang Y. Depletion of residual amoxicillin and its major metabolites in muscle. Liver and Kidney Chicken PVJ. (2019) 39:19–24. doi: 10.29261/pakvetj/2018.120

[27] European Medicines Agency (EMA). EMA - AMEG classification: Categorisation of antibiotics in the European Union. (2025). Available at: https://www.ema.europa.eu/en/documents/report/categorisation-antibiotics-european-union-answer-request-european-commission-updating-scientific-advice-impact-public-health-animal-health-use-antibiotics-animals_en.pdf (accessed October 7, 2025)

[28] World Health Organization (WHO). WHO - AWaRe classification: WHO AWaRe classification database of antibiotics for evaluation and monitoring of use. (2023). Available at: https://www.who.int/publications/i/item/WHO-MHP-HPS-EML-2023.04 (accessed October 7, 2025)

[29] Wang G Wang B Zhao X Xie X Xie K Wang X. Determination of thiamphenicol, florfenicol and florfenicol amine residues in poultry meat and pork via ASE-UPLC-FLD. J Food Compos Anal. (2019) 81:19–27. doi: 10.1016/j.jfca.2019.05.004

[30] Mosleh N Shomali T Namazi F Marzban M Mohammadi M Boroojeni AM. Comparative evaluation of therapeutic efficacy of sulfadiazine-trimethoprim, oxytetracycline, enrofloxacin and florfenicol onStaphylococcus aureus–induced arthritis in broilers. Br Poult Sci. (2016) 57:179–84. doi: 10.1080/00071668.2016.1148263, PMID: 27111299

[31] Laconi A Tolosi R Mughini-Gras L Cuccato M Cannizzo FT Piccirillo A. Amoxicillin and thiamphenicol treatments may influence the co-selection of resistance genes in the chicken gut microbiota. Sci Rep. (2022) 12:20413. doi: 10.1038/S41598-022-24927-7, PMID: 36437351 PMC9701756

[32] Serrano MJ Mitjana O Bonastre C Laborda A Falceto MV García-Gonzalo D. Is blood a good Indicator for detecting antimicrobials in meat? Evidence for the development of in vivo surveillance methods. Antibiotics. (2020) 9:175. doi: 10.3390/antibiotics9040175, PMID: 32290542 PMC7235904

[33] Meklati FR Panara A Hadef A Meribai A Ben-Mahdi MH Dasenaki ME. Comparative assessment of antibiotic residues using liquid chromatography coupled with tandem mass spectrometry (LC-MS/MS) and a rapid screening test in raw milk collected from the north-central Algerian dairies. Toxics. (2022) 10:19. doi: 10.3390/toxics10010019, PMID: 35051061 PMC8781432

[34] Bao Y Li Y Cai K Liu Y Li B. Rapid detection of antibiotics using self-developed electrochemical analyzer and sensor chip. Chem Commun. (2025) 61:11211–4. doi: 10.1039/D5CC02741A, PMID: 40553488

