# Supplementary material for: Rapid detection of antimicrobial residues in broiler meat using flow cytometry-driven multiplex immunoassay
Source: Front Vet Sci. 2025 Oct 30;12:1636223. doi: 10.3389/fvets.2025.1636223 (PMC12613232; doi:10.3389/fvets.2025.1636223)
Supplement: Supplementary file 1 [file Table_1.docx]

**Tab. S1** nMFI values of each animal of control group and calculated LOD values for thiamphenicol (T), amoxicillin (A) and sulphadiazine (S).

|  | nMFI | | |
| --- | --- | --- | --- |
| **Case n.** | **T** | **A** | **S** |
| **1** | 92.75 | 90.70 | 79.79 |
| **2** | 74.86 | 80.75 | 71.55 |
| **3** | 90.86 | 95.75 | 71.94 |
| **4** | 70.11 | 80.69 | 68.46 |
| **5** | 76.82 | 91.49 | 64.62 |
| **6** | 82.38 | 79.21 | 71.33 |
| **7** | 81.15 | 90.12 | 82.75 |
| **8** | 92.96 | 85.31 | 66.87 |
| **9** | 112.96 | 96.76 | 93.78 |
| **10** | 99.07 | 94.84 | 67.09 |
| **11** | 98.31 | 85.69 | 75.87 |
| **12** | 85.06 | 85.37 | 64.40 |
| Mean | 88.11 | 88.06 | 73.21 |
| SD | 12.10 | 6.11 | 8.65 |
| LOD | 51.79 | 69.74 | 47.24 |

**Tab. S2** nMFI values for thiamphenicol obtained in each animal of the thiamphenicol (T). amoxicillin (A) and sulphadiazine (S) group treatment

|  | TIAMPHENICOL | | |  |
| --- | --- | --- | --- | --- |
| Case n. | Group | nMFI | Test result* | HPLC-HRMS |
| 13 | A | 62.67 | NEG | NEG |
| 14 | A | 65.36 | NEG | NEG |
| 15 | A | 47.23 | POS | NEG |
| 16 | A | 17.68 | POS | NEG |
| 17 | A | 75.19 | NEG | POS |
| 18 | A | 60.79 | NEG | N.D. |
| 19 | A | 64.53 | NEG | NEG |
| 20 | A | 62.82 | NEG | POS |
| 21 | A | 88.83 | NEG | NEG |
| 22 | A | 59.17 | NEG | NEG |
| 23 | A | 60.58 | NEG | NEG |
| 24 | A | 83.69 | NEG | NEG |
| 25 | S | 98.93 | NEG | NEG |
| 26 | S | 69.91 | NEG | NEG |
| 27 | S | 30.33 | POS | NEG |
| 28 | S | 99.56 | NEG | NEG |
| 29 | S | 79.52 | NEG | NEG |
| 30 | S | 78.23 | NEG | NEG |
| 31 | T | 27.90 | POS | POS |
| 32 | T | 24.11 | POS | POS |
| 33 | T | 28.70 | POS | POS |
| 34 | T | 20.13 | POS | POS |
| 35 | T | 23.04 | POS | POS |
| 36 | T | 23.54 | POS | N.D. |
| 37 | T | 16.84 | POS | POS |
| 38 | T | 17.96 | POS | POS |
| 39 | T | 26.49 | POS | POS |
| 40 | T | 20.45 | POS | POS |
| 41 | T | 21.70 | POS | POS |

* Interpreted according to a nMFI greater than or less than the LOD value. POS: positive result (nMFI < 51.79); NEG: negative result (nMFI ≥ 51.79)

N.D.: not determined

|  | AMOXICILLIN | | |  |
| --- | --- | --- | --- | --- |
| Case n. | Group | nMFI | Test result* | HPLC-HRMS |
| 13 | A | 117.04 | NEG | NEG |
| 14 | A | 97.70 | NEG | NEG |
| 15 | A | 95.18 | NEG | NEG |
| 16 | A | 85.52 | NEG | NEG |
| 17 | A | 103.24 | NEG | NEG |
| 18 | A | 85.49 | NEG | N.D. |
| 19 | A | 85.30 | NEG | NEG |
| 20 | A | 79.89 | NEG | NEG |
| 21 | A | 94.85 | NEG | NEG |
| 22 | A | 94.15 | NEG | NEG |
| 23 | A | 83.66 | NEG | POS |
| 24 | A | 87.48 | NEG | NEG |
| 25 | S | 84.81 | NEG | NEG |
| 26 | S | 70.84 | NEG | NEG |
| 27 | S | 81.11 | NEG | NEG |
| 28 | S | 90.47 | NEG | NEG |
| 29 | S | 84.42 | NEG | NEG |
| 30 | S | 92.67 | NEG | NEG |
| 31 | T | 109.25 | NEG | NEG |
| 32 | T | 79.89 | NEG | NEG |
| 33 | T | 77.90 | NEG | NEG |
| 34 | T | 41.36 | NEG | NEG |
| 35 | T | 92.47 | NEG | NEG |
| 36 | T | 84.73 | NEG | N.D. |
| 37 | T | 67.25 | NEG | NEG |
| 38 | T | 82.20 | NEG | NEG |
| 39 | T | 77.89 | NEG | NEG |
| 40 | T | 84.74 | NEG | NEG |
| 41 | T | 63.58 | NEG | NEG |

**Tab. S3** nMFI values for amoxicillin obtained in each animal of the thiamphenicol (T). amoxicillin (A) and sulphadiazine (S) group treatment

* Interpreted according to a nMFI greater than or less than the LOD value. POS: positive result (nMFI < 69.74); NEG: negative result (nMFI ≥ 69.74)

N.D.: not determined.

**Tab. S4** nMFI values for sulphadiazine obtained in each animal of the thiamphenicol (T). amoxicillin (A) and sulphadiazine (S) group treatment

|  | SULPHADIAZINE | | |  |
| --- | --- | --- | --- | --- |
| **Case n.** | **Group** | **nMFI** | **Test result** | HPLC-HRMS |
| 13 | A | 88.79 | NEG | NEG |
| 14 | A | 66.08 | NEG | NEG |
| 15 | A | 72.74 | NEG | NEG |
| 16 | A | 67.16 | NEG | NEG |
| 17 | A | 78.71 | NEG | NEG |
| 18 | A | 70.10 | NEG | N.D. |
| 19 | A | 70.14 | NEG | NEG |
| 20 | A | 69.75 | NEG | NEG |
| 21 | A | 82.36 | NEG | NEG |
| 22 | A | 83.23 | NEG | NEG |
| 23 | A | 69.50 | NEG | NEG |
| 24 | A | 71.16 | NEG | NEG |
| 25 | S | 5.26 | POS | POS |
| 26 | S | 3.11 | POS | POS |
| 27 | S | 3.15 | POS | POS |
| 28 | S | 5.60 | POS | POS |
| 29 | S | 3.91 | POS | POS |
| 30 | S | 5.39 | POS | POS |
| 31 | T | 88.01 | NEG | NEG |
| 32 | T | 64.34 | NEG | NEG |
| 33 | T | 69.74 | NEG | NEG |
| 34 | T | 41.60 | POS | NEG |
| 35 | T | 79.80 | NEG | NEG |
| 36 | T | 70.59 | NEG | N.D. |
| 37 | T | 45.35 | POS | NEG |
| 38 | T | 62.68 | NEG | NEG |
| 39 | T | 78.29 | NEG | NEG |
| 40 | T | 61.75 | NEG | NEG |
| 41 | T | 56.31 | NEG | NEG |

* Interpreted according to a nMFI greater than or less than the LOD value. POS: positive result (nMFI < 47.24); NEG: negative result (nMFI ≥ 47.24)

N.D.: not determined.
